# Combination therapy with irinotecan and cisplatin as neoadjuvant chemotherapy in locally advanced cervical cancer

**DOI:** 10.1038/sj.bjc.6690656

**Published:** 1999-09

**Authors:** T Sugiyama, T Nishida, S Kumagai, S Nishio, K Fujiyoshi, N Okura, M Yakushiji, M Hiura, N Umesaki

**Affiliations:** Department of Obstetrics and Gynecology, Kurume University School of Medicine, Kurume City, 830-0011, Japan; National Shikoku Cancer Center, 13 Horinouchi, Matsuyama City, Japan; Osaka City University, 1-5-7 Asahi-machi, Osaka City, 545-0051, Japan

**Keywords:** CPT-11, cisplatin, cervical cancer, squamous cell carcinoma, neoadjuvant chemotherapy

## Abstract

To evaluate the response rate and toxicity of the combination of irinotecan (CPT-11) and cisplatin in a neoadjuvant setting, a phase II study was conducted regarding the regimen of this combination in patients with locally advanced cervical cancer. Eligibility included patients with previously untreated stage Ib2, IIb, or IIIb squamous cell carcinoma with good performance status. CPT-11 (60 mg m^−2^) was administered intravenously on days 1, 8 and 15, followed by cisplatin (60 mg m^−2^) given intravenously on day 1. Treatment was repeated every 4 weeks for a total of two or three cycles. Among 23 eligible patients (median age: 59 years), three showed complete response (13%), 15 showed partial response (65%), for an overall response rate of 78% (95% confidence interval 58–90%). Stable disease was observed in four cases (17%) and progressive disease in one (4%). The median time to failure and median survival time have not yet been reached. Of the 52 treatment cycles administered, diarrhoea and grade 3 or 4 neutropenia were observed in 10% and 75% respectively. There were no therapy-related deaths. The combination of CPT-11 with cisplatin is a promising regimen for neoadjuvant chemotherapy in locally advanced cervical cancer. The toxicities of this regimen are well tolerated. © 1999 Cancer Research Campaign


British Journal of Cancer (1999) 81(1), 95–98
© 1999 Cancer Research Campaign
Article no. bjoc.1999.0656

### Combination therapy with irinotecan and cisplatin as neoadjuvant chemotherapy in locally advanced cervical cancer

**T Sugiyama[1], T Nishida[1], S Kumagai[1], S Nishio[1], K Fujiyoshi[1], N Okura[1], M Yakushiji[1], M Hiura[2] and N Umesaki[3]**

[1]Department of Obstetrics and Gynecology, Kurume University School of Medicine, 67 Asahi-machi, Kurume City, 830-0011, Japan; [2]National Shikoku Cancer Center, 13 Horinouchi, Matsuyama City, 790-0007, Japan; [3]Osaka City University, 1-5-7 Asahi-machi, Abeno-ku, Osaka City, 545-0051, Japan

**Summary** To evaluate the response rate and toxicity of the combination of irinotecan (CPT-11) and cisplatin in a neoadjuvant setting, a phase II study was conducted regarding the regimen of this combination in patients with locally advanced cervical cancer. Eligibility included patients with previously untreated stage Ib2, IIb, or IIIb squamous cell carcinoma with good performance status. CPT-11 (60 mg m$^{-2}$) was administered intravenously on days 1, 8 and 15, followed by cisplatin (60 mg m$^{-2}$) given intravenously on day 1. Treatment was repeated every 4 weeks for a total of two or three cycles. Among 23 eligible patients (median age: 59 years), three showed complete response (13%), 15 showed partial response (65%), for an overall response rate of 78% (95% confidence interval 58–90%). Stable disease was observed in four cases (17%) and progressive disease in one (4%). The median time to failure and median survival time have not yet been reached. Of the 52 treatment cycles administered, diarrhoea and grade 3 or 4 neutropenia were observed in 10% and 75% respectively. There were no therapy-related deaths. The combination of CPT-11 with cisplatin is a promising regimen for neoadjuvant chemotherapy in locally advanced cervical cancer. The toxicities of this regimen are well tolerated.

**Keywords:** CPT-11; cisplatin; cervical cancer; squamous cell carcinoma; neoadjuvant chemotherapy

Advanced cervical cancer is the fifth most common cancer worldwide and is the second major cause of death in women (Parkin et al, 1988). Studies on the use of cisplatin have shown that squamous cell carcinoma of the uterine cervix is sensitive to chemotherapy (Thigpen et al, 1981; Alberts et al, 1987). Various cisplatin-based regimens have been associated with high responses in patients with advanced and recurrent cervical cancers (Lahousen et al, 1987; Omura et al, 1992; Thigpen et al, 1995). However, only a few well-designed randomized studies have been carried out, and chemotherapy in patients with refractory cervical cancer usually results in excessive toxicity and a short duration of response with no survival advantage. In recent years, cisplatin-containing chemotherapy has been used as neoadjuvant chemotherapy (NAC) to treat patients with International Federation of Gynecology and Obstetrics (FIGO) stage IIb, III, or IV diseases as well as stage Ib or IIa disease with a bulky mass. NAC has been reported as useful in the control of local or regional disease, and is useful in controlling remote metastasis (Benedetti-Panici et al, 1991; Souhami et al, 1991; Vermorken, 1993; Scarabelli et al, 1995; Sundfor et al, 1996; Sugiyama et al, 1998*a*). Recently, NAC followed by radical surgery has been proposed to improve the survival rate of patients with locally advanced cervical cancer (Benedetti-Panici et al, 1991; Scarabelli et al, 1995; Sugiyama et al, 1998*a*). Because the optimal NAC regimen has only recently been established, we need to develop a new combination chemotherapy regimen to achieve clinical benefits in the treatment of locally advanced cervical cancer.

Irinotecan hydrochloride (CPT-11) is a semisynthetic derivative of camptothecin, a plant alkaloid obtained from *Camptotheca acuminata* (Wall et al, 1996), which is capable of potent anti-tumour activity. The anti-tumour effects of CPT-11 are related to the inhibition of DNA topoisomerase I (Kunimoto et al, 1987; Hsiang et al, 1989), a novel mechanism among other anti-tumour agents. CPT-11 expresses a potent activity against various experimental tumour models (Matsuzaki et al, 1988) and shows slow cross-resistance to other anti-tumour agents (Tsuruo et al, 1988). It has also been found in some clinical trials to be clinically active against various cancers including cervical cancer (Takeuchi et al, 1991; Verschraegen et al, 1997; Irvin et al, 1998). Moreover, the combination of CPT-11 and cisplatin, which has shown a marked synergism experimentally (Itoh et al, 1991; Kano et al, 1992; Minagawa et al, 1994), has been reported to be effective, with acceptable toxicity, against advanced or recurrent cervical cancer (Sugiyama et al, 1995; Sugiyama et al, 1998*b*). In a phase I study of a combination of CPT-11 with cisplatin, in which cisplatin was administered at the recommended dose of 60 mg m$^{-2}$ on day 1, CPT-11 was administered at doses of 60 mg m$^{-2}$ on days 1, 8 and 15 (Sugiyama et al, 1995).

We conducted a phase II study of CPT-11 administered in combination with cisplatin to patients with locally advanced cervical cancer. The objectives of this study were to determine the efficacy and safety of the combination as a neoadjuvant chemotherapy.

*Received 22 September 1998*
*Revised 25 March 1999*
*Accepted 12 April 1999*

*Correspondence to:* T Sugiyama

### MATERIALS AND METHODS

### Patient selection

Previously untreated patients with stage Ib, IIb, or IIIb squamous cell carcinoma of the cervix were enrolled. Eligibility criteria were as follows: age ≤ 75 years, World Health Organization (WHO) Performance Status (PS) of ≤ 2, adequate bone marrow reserve (leukocyte count: ≥ 4 × $10^3$ $\mu l^{-1}$, platelet count: ≥ 100 × $10^3$ $\mu l^{-1}$), and adequate renal and hepatic function (serum creatinine: <1.5 mg $dl^{-1}$, creatinine clearance: ≥ 50 ml $min^{-1}$, BUN: < 25 mg $dl^{-1}$, AST and ALT: ≤ twice the upper normal limit, and serum total bilirubin: < 1.5 mg $dl^{-1}$). All patients gave their written informed consent for participation.

### Treatment design

We designed the dose and treatment schedule of cisplatin and CPT-11 on the basis of our phase I study in patients with recurrent or advanced cervical cancer (Sugiyama et al, 1995). An intravenous (i.v.) infusion of over 60 min of CPT-11 60 mg $m^{-2}$ was given on days 1, 8 and 15. On completion of CPT-11 infusion on day 1, cisplatin at 60 mg $m^{-2}$ was administered i.v. for over 90 min. Before and after cisplatin administration, patients received i.v. hydration with 1500–2000 ml 0.9% normal saline or 5% dextrose. A 5-HT3 serotonin receptor antagonist was administered according to the attending physician's judgment. Granulocyte colony-stimulating factor (G-CSF) was administered if grade 3 neutropenia with fever (≥ 38.0°C) or grade 4 neutropenia developed. This treatment schedule was repeated every 4 weeks for two or three cycles. Following chemotherapy, radical surgery was performed in all patients with stages Ib2 and IIb disease who were younger than 70. With regard to IIIb disease, patients underwent radical surgery only if they responded to chemotherapy, and if cancer-free space between the tumour and the pelvic wall was recognized on rectal examination at the completion of NAC. Patients with stage IIIb disease who did not respond to chemotherapy underwent radiotherapy.

The dose levels and treatment schedule were modified to avoid severe side-effects. CPT-11 was not given on days 8 and/or 15 if the patient's leukocyte or platelet counts were less than 3.0 × $10^3$ $\mu l^{-1}$ or less than 75 × $10^3$ $\mu l^{-1}$ respectively. It was also withheld if the patient developed diarrhoea of grade 2 or worse according to the Eastern Cooperative Oncology Group (ECOG) scale (Oken et al, 1982). Before the next cycle was started, a leukocyte count ≥ 3.0 × $10^3$ $\mu l^{-1}$, and platelet count ≥ 100 × $10^3$ $\mu l^{-1}$, with no diarrhoea observed, and liver and renal function according to the eligibility criteria had to be satisfied. Additionally, if ≥ grade 3 diarrhoea during any cycle was observed, the CPT-11 dose in the next cycle was reduced to 50 mg $m^{-2}$. If serum creatinine level showed 1.5 times the upper normal limit, the cisplatin dose was reduced to 50 mg $m^{-2}$.

The trial was approved by the Institutional Review Board of the Clinical Oncology Program at each hospital.

### Response evaluation

Cervical tumour measurements using magnetic resonance imaging (MRI) were obtained at the completion of every treatment cycle. Measurement of response was based on the product of the two largest perpendicular diameters. Criteria for tumour response were as follows: complete response (CR) was defined as the complete disappearance of all known disease for a minimum of 4 weeks, with no development of new disease. Partial response (PR) was defined as a ≥ 50% reduction of the sum of products of measurable lesions for a minimum of 4 weeks with no development of new lesions. Progressive disease (PD) was defined as a ≥ 25% increase in the sum of the products of all indicator lesions, or reappearance of any lesion that had disappeared, or appearance of any new lesion. Stable disease (SD) was defined by any situation that did not qualify as response or progression. Serum squamous cell carcinoma-associated antigen (SCC) level was measured using a commercially available kit (Abbott Laboratories, Diagnostic Division, Silic 203, France) at the completion of every treatment cycle.

Toxicity evaluation was based on WHO criteria (WHO offset publication, 1979), except for diarrhoea, which was based on Eastern Cooperative Oncology Group (ECOG) criteria (Oken et al, 1982). Complete blood cell counts were performed at least twice weekly. Serum chemistries and liver function tests were obtained before every treatment cycle.

### RESULTS

Between May 1996 and April 1998, 23 patients with locally advanced squamous cell cervical cancer who had no prior therapy were enrolled from the three cooperating institutions. The patients' median age was 59 years (range 23–75 years). Nineteen patients (83%) had a PS of 0, and four patients (17%) a PS of 1. Two patients (9%) had stage Ib2 disease, eight (35%) had stage IIb disease with bulky mass (≥ 4 cm) and 13 patients (57%) had stage IIIb disease. All patients are assessable for response and toxicity. Seventeen patients (74%) received two cycles of therapy, and the remaining six patients received three cycles of therapy (Table 1).

### Response to therapy

There were three CRs (13%) and 15 PRs (65%), with an overall response rate of 78% (95% confidence interval (CI) 58–90%). SD was observed in four patients (17%), while PD was recorded in only one (4%) patient. Table 2 lists results according to FIGO stage. Twenty patients (87%) had positive serum SCC levels (≥ 1.5 ng $ml^{-1}$) before treatment. SCC level fell to negative in all

**Table 1** Patient characteristics

| Characteristic | No. of patients | % |
|---|---|---|
| Entered on study | 23 | 100 |
| Age (years) | | |
| Median | 59 | |
| Range | 23–75 | |
| Performance status | | |
| 0 | 19 | 83 |
| 1 | 4 | 17 |
| FIGO stage[a] | | |
| Ib2 | 2 | 9 |
| IIb | 8 | 35 |
| IIIb | 13 | 57 |
| No. of cycles | | |
| 2 | 17 | 74 |
| 3 | 6 | 26 |

[a]International Federation of Gynecology and Obstetrics (FIGO).

© 1999 Cancer Research Campaign

**Table 2** Results according to stage

| Stage[a] | No. of patients | Response | | | |
|---|---|---|---|---|---|
| | | CR | PR | SD | PD |
| Ib2 | 2 | 0 | 1 | 1 | 0 |
| IIb | 8 | 1 | 6 | 1 | 0 |
| IIIb | 13 | 2 | 8 | 2 | 1 |
| Total (%) | 23 | 3 (13) | 15 (65) | 4 (17) | 1 (4) |

CR, complete response; PR, partial response; SD, stable disease; PD, progressive disease. [a]Stage, International Federation of Gynecology and Obstetrics (FIGO).

**Table 3** Toxicity[a] (per treatment cycles, $n = 52$)

| | Grade 1 | | Grade 2 | | Grade 3 | | Grade 4 | |
|---|---|---|---|---|---|---|---|---|
| | No. | % | No. | % | No. | % | No. | % |
| Haematological | | | | | | | | |
| Anaemia | 13 | 25 | 16 | 31 | 14 | 27 | 0 | 0 |
| Leukopenia | 14 | 27 | 23 | 44 | 10 | 19 | 4 | 8 |
| Neutropenia | 1 | 2 | 12 | 23 | 29 | 56 | 10 | 19 |
| Thrombocytopenia | 3 | 6 | 2 | 4 | 0 | 0 | 0 | 0 |
| Gastrointestinal | | | | | | | | |
| Nausea/vomiting | 14 | 27 | 22 | 42 | 7 | 13 | 1 | 2 |
| Liver toxicity | 3 | 6 | 1 | 2 | 0 | 0 | 0 | 0 |
| Diarrhoea | 13 | 25 | 13 | 25 | 2 | 4 | 3 | 6 |
| Renal toxicity | 8 | 15 | 2 | 4 | 0 | 0 | 0 | 0 |
| Alopecia[b] | 15 | 65 | 6 | 26 | – | – | – | – |

[a]World Health Organization (WHO) criteria. [b]Per patient ($n = 23$).

three patients with CR. Among 13 patients with PR, the level fell to negative in ten patients (77%) and decreased in three patients (23%). Among three patients with SD, the level fell to negative in one patient and decreased in the remaining two patients. However, the level was elevated in one patient with PD.

Among ten cases of Ib and IIb stage, radical surgery was performed after NAC in nine cases (younger than 70) with the patient's consent, and radiotherapy was performed for the remaining one patient who was older than 70. In ten patients with stage IIIb disease who responded to NAC, their symptomatic stage improved and radical surgery was considered to be possible, so radical surgery was performed for five patients. Radiotherapy was performed in the three patients whose informed consent for operation was not obtained and in the remaining two patients of advanced age (> 70 years). Radiotherapy was also performed in three patients of SD or PD to NAC.

### Toxicity

The major toxicity was neutropenia, of which grade 3 or 4 severity developed in 29 treatment cycles (56%) or ten treatment cycles (19%) of a total of 52 treatment cycles respectively. Grade 2 infection occurred in two cycles (4%). G-CSF was administered for 2–6 days during 11 (21%) of the 52 treatment cycles. Thrombocytopenia was less common, and grade 3 or 4 thrombocytopenia was not recorded. Grade 3 anaemia occurred in 14 of a total of 52 cycles; grade 4 anaemia was not observed. Two patients (9%) required a total 5 U of packed RBCs because of consecutive radical surgeries.

The second major toxicity was diarrhoea, which developed in 31 cycles (60%) of a total of 52 cycles, though grade 3 or 4 diarrhoea was observed in just five cycles (10%). Diarrhoea diminished with administration of loperamide hydrochloride and/or i.v. drop infusion of electrolyte for 2–3 days. Nausea and vomiting was also a common expression of non-haematologic toxicity, and grade 2 severity or above was recorded in 30 cycles (58%). Alopecia was observed in 20 patients (91%), in which grade 2 severity was 50%. Other adverse effects such as neurological toxicity, dermatitis, stomatitis, or allergic reactions were not observed (Table 3).

While CPT-11 administration was omitted on days 8 or 15 in 16 (31%) of 52 treatment cycles because of the presence of ≥ grade 2 diarrhoea and/or neutropenia (leukopenia), a dose reduction was required in only 3 cycles (6%), and a > 1 week treatment delay was observed in only 2 cycles (4%). There were no treatment-related deaths.

### DISCUSSION

In addition to cisplatin, bleomycin, 5-fluorouracil, mitomycin C, ifosfamide, vincristine, adriamycin and CPT-11 are also active against uterine cervical cancer (Thigpen et al, 1981, 1995; Alberts et al, 1987; Lahousen et al, 1987; Takeuchi et al, 1991; Omura et al, 1992; Verschraegen et al, 1997; Irvin et al, 1998). In a previous phase II study of the effect of CPT-11 alone on recurrent cervical cancer in Japan, CPT-11 was given at a dose of 100 mg $m^{-2}$ four times with 1-week intervals and at a dose of 150 mg $m^{-2}$ three times with 2-week intervals (Takeuchi et al, 1991). The response rate was 23.6%, and there was no significant difference between the two schedules. The major toxicities were leukopenia and diarrhoea in both schedules. In a phase II study of patients who had previously undergone chemotherapy in the USA, CPT-11 was given at a dose of 125 mg $m^{-2}$ four times with 1-week intervals, and the response rate, 21%, was similar to the results in Japan (Verschraegen et al, 1997). It was thus recognized that CPT-11 is effective against cervical cancer.

There is reportedly no cross-resistance but a synergistic effect in vitro between CPT-11 and cisplatin (Tsuruo et al, 1988; Kano et al, 1992; Minagawa et al, 1994). In a phase I study of a combination of CPT-11 with cisplatin, in which cisplatin was administered at the recommended dose of 60 mg $m^{-2}$ on day 1, CPT-11 was administered at doses of 60 mg $m^{-2}$ on days 1, 8 and 15 (Sugiyama et al, 1995). Recently, a phase II study of the combination of CPT-11 and cisplatin based on the results of the phase I study in patients with advanced and recurrent cervical cancer was completed and the effectiveness and safety of this regimen have been described (Sugiyama et al, 1998*b*). We conducted the present study in a neoadjuvant setting at the same time. Neutropenia was the dose-limiting toxicity but was reversed by treatment with G-CSF or by omitting CPT-11 administration on day 8 or 15. Thrombocytopenia was infrequent and less severe. Diarrhoea developed in 60% of the cycles, but it was mild and was controlled with the use of antidiarrhoeal agents, such as loperamide. Prophylactic and/or therapeutic administration of a 5-HT3 serotonin receptor antagonist reduced the severity of nausea and vomiting.

Although high response rates induced by NAC have been reported by many investigators, it is difficult to compare the results of these reports because of the wide variation in the patients' characteristics. In general, the response rate has been reported to be around 70% for NAC with a cisplatin-based

© 1999 Cancer Research Campaign regimen (Benedetti-Panici et al, 1991; Souhami et al, 1991; Vermorken, 1993; Scarabelli et al, 1995; Sundfor et al, 1996; Sugiyama et al, 1998*a*). In this study, we observed a response rate of 78%. These results can be favourably compared with the values described, and toxicity could be controlled.

In conclusion, combination therapy with CPT-11 and cisplatin appeared to be highly active and improve the operability rate against previously untreated uterine cervical cancer over short periods of time. Its toxicity is acceptable, and the regimen is worth consideration in respect to its effect of extending the survival time in cases undergoing NAC followed by radical surgery for progressive stage cervical cancers.

### REFERENCES

Alberts DS, Kronmal R and Baker LH (1987) Phase II randomized trial of cisplatin chemotherapy of recurrent or metastatic squamous cell carcinoma of the cervix: a Southwest Oncology Group Study. *J Clin Oncol* **5**: 1791–1795

Benedetti-Panici P, Greggi S, Scambia G, et al. (1991) High-dose cisplatin and bleomycin neoadjuvant chemotherapy plus radical surgery in locally advanced cervical carcinoma: a preliminary report. *Gynecol Oncol* **41**: 212–216

Hsiang YH, Liu LF, Wall ME, et al (1989) DNA topoisomerase I mediated DNA cleavage and cytotoxicity of camptothecin analogues. *Cancer Res* **49**: 4835–4839

Irvin WP, Price FV, Bailey H, et al (1998) A phase II study of irinotecan (CPT-11) on patients with advanced squamous cell carcinoma of the cervix. *Cancer* **82**: 328–333

Kano Y, Suzuki K, Akutsu M, et al (1992) Effects of CPT-11 in combination with other anticancer agents in culture. *Int J Cancer* **50**: 604–610

Kunimoto T, Nitta K, Tanaka T, et al (1987) Antitumor activity of 7-ethyl-10-[4-(1-piperidino)-10-piperidino]carbonyloxy-camptothecin, a novel water-soluble derivative of camptothecin, against murine tumors. *Cancer Res* **47**: 5944–5947

Lahousen M, Pickel H and Tamussino K (1987) Chemotherapy for advanced and/or recurrent cervical cancer. *Arch Gynecol* **240**: 247–252

Matsuzaki T, Yokokura T, Mutai M, et al (1988) Inhibition of spontaneous and experimental metastasis by a new derivative of camptothecin, CPT-11, in mice. *Cancer Chemother Pharmacol* **21**: 308–312

Minagawa Y, Kigawa J, Ishikawa H, et al (1994) Synergistic enhancement of cisplatin cytotoxicity by SN-38, an active metabolite of CPT-11, for cisplatin-resistant HeLa cells. *Jpn J Cancer Res* **85**: 966–971

Oken MM, Creech RH, Tormey DC, et al. (1982) Toxicity and response criteria of the Eastern Cooperative Oncology Group. *Am J Clin Oncol* **5**: 649–655

Omura GA (1992) Current status of chemotherapy for cancer of the cervix. *Oncology* **6**: 27–32

Parkin DM, Laara E and Muir CS (1988) Estimates of the worldwide frequency of sixteen major cancers in 1980. *Int J Cancer* **41**: 184–197

Scarabelli C, Zarrelli A, Gallo A and Visentin MC (1995) Multimodal treatment with neoadjuvant intraarterial chemotherapy and radical surgery in patients with stage IIIb–IVa cervical cancer. A preliminary study. *Cancer* **76**: 1019–1026

Souhami L, Gil RA, Allan SE, et al. (1991) A randomized trial of chemotherapy followed by pelvic radiation therapy in stage IIIB carcinoma of the cervix. *J Clin Oncol* **9**: 970–977

Sugiyama T, Takeuchi S, Noda K, et al (1995) Phase I study of irinotecan (CPT-11) in combination with cisplatin in cervical cancer. *Proc Am Soc Clin Oncol* **13**: A1546

Sugiyama T, Nishida T, Hasuo Y, Fujiyoshi K and Yakushiji M (1998*a*) Neoadjuvant intraarterial chemotherapy followed by radical hysterectomy and/or radiotherapy for locally advanced cervical cancer. *Gynecol Oncol* **69**: 130–136

Sugiyama T, Noda K, Yakushiji M, et al (1998*b*) Phase II study of irinotecan (CPT-11) in combination with cisplatin in cervical cancer. *Proc Am Soc Clin Oncol* **17**: 1360

Sundfor K, Trope CG, Hogberg T, et al. (1996) Radiotherapy and neoadjuvant chemotherapy for cervical carcinoma. A randomized multicenter study of sequential cisplatin and 5-fluorouracil and radiotherapy in advanced cervical carcinoma stage 3B and 4A. *Cancer* **77**: 2371–2378

Takeuchi S, Dobashi K, Fujimoto S, et al (1991) A late phase II study of CPT-11 on uterine cervical cancer and ovarian cancer. *Gan To Kagaku Ryoho* **18**: 1681–1698

Thigpen T, Shingleton H, Homesley H, Lagasse L and Blessing J (1981) Cis-platinum in treatment of advanced or recurrent squamous cell carcinoma of the cervix: a phase II study of the Gynecologic Oncology Group. *Cancer* **48**: 899–903

Thigpen T, Vance R, Puneky L and Khansur T (1995) Chemotherapy as a palliative treatment in carcinoma of the uterine cervix. *Semin Oncol* **22**: 16–24

Tsuruo T, Matsuzaki T, Matsushita M, et al (1988) Antitumor effect of CPT-11, a new derivative of camptothecin, against pleiotropic drug-resistant tumor in vitro and vivo. *Cancer Chemother Pharmacol* **21**: 71–74

Vermorken JB (1993) The role of chemotherapy in squamous cell carcinoma of the uterine cervix: a review. *Int J Gynecol Cancer* **3**: 129–142

Verschraegen CF, Levy T, Kudelka AP, et al (1997) Phase II study of irinotecan in prior chemotherapy-treated squamous cell carcinoma of the cervix. *J Clin Oncol* **15**: 625–631

Wall ME, Wani MC, Cook CE, et al (1996) Plant antitumor agents. I. The isolation and structure of camptothecin, a novel alkaloidal leukemia and tumor inhibitor from *Camptotheca acuminata*. *J Am Chem Soc* **88**: 3888–3890

WHO Handbook for Reporting Results of Cancer Treatment (1979) WHO Offset Publication No. 48. World Health Organization: Geneva

*© 1999 Cancer Research Campaign*
